# Sex-specific ecophysiological responses to environmental fluctuations of free-ranging Hermann's tortoises: implication for conservation

**DOI:** 10.1093/conphys/cow054

**Published:** 2016-11-09

**Authors:** Adélaïde Sibeaux, Catherine Louise Michel, Xavier Bonnet, Sébastien Caron, Kévin Fournière, Stephane Gagno, Jean-Marie Ballouard

**Affiliations:** 1 CRCC Centre for Research and Conservation of Chelonians, SOPTOM, Var, 83590 Gonfaron, France; 2 CIE Centre for Integrative Ecology, School of Life and Environmental Sciences, Deakin University, 3125 Victoria, Australia; 3 Centre d'Etudes Biologiques de Chizé, CEBC UMR-7372, CNRS-Université de La Rochelle, 79360 Villiers en bois, France

**Keywords:** Body condition, corticosterone, population managment, reptile conservation, translocation methodology

## Abstract

Physiological parameters provide indicators to evaluate how organisms respond to conservation actions. For example, individuals translocated during reinforcement programmes may not adapt to their novel host environment and may exhibit elevated chronic levels of stress hormones and/or decreasing body condition. Conversely, successful conservation actions should be associated with a lack of detrimental physiological perturbation. However, physiological references fluctuate over time and are influenced by various factors (e.g. sex, age, reproductive status). It is therefore necessary to determine the range of natural variations of the selected physiological metrics to establish useful baselines. This study focuses on endangered free-ranging Hermann's tortoises (*Testudo hermanni hermanni*), where conservation actions have been preconized to prevent extinction of French mainland populations. The influence of sex and of environmental factors (site, year and season) on eight physiological parameters (e.g. body condition, corticosterone concentrations) was assessed in 82 individuals from two populations living in different habitats. Daily displacements were monitored by radio-tracking. Most parameters varied between years and seasons and exhibited contrasting sex patterns but with no or limited effect of site. By combining behavioural and physiological traits, this study provides sex-specific seasonal baselines that can be used to monitor the health status of Hermann's tortoises facing environmental threats (e.g. habitat changes) or during conservation actions (e.g. translocation). These results might also assist in selection of the appropriate season for translocation.

## Abstract (precis)

We assessed the range of variation of physiological indicators (e.g. glucocorticoid) and movements in the endangered Hermann's tortoises. The findings provide sex-specific seasonal baselines that can be used to monitor the health status of tortoises facing environmental threats or during conservation actions (e.g. translocation).

## Introduction

Understanding the physiological responses of organisms to environmental changes can improve conservation strategies (Wikelski and Cooke, 2006). Indeed, even closely related species exhibit different triggering factors and different physiological limits to external fluctuations, and these divergences determine their respective adaptability to changing conditions (Helmuth *et al*., 2005). Relevant physiological mechanisms should be scrutinized in each species to forecast population responses to global changes (Kearney and Porter, 2009). Furthermore, the intensity, appropriateness or failure of the responses to external conditions depends on the physiological status of each individual at a given time. For example, in female reptiles, body condition at the onset of the breeding season influences the decision to reproduce, the mobilization of maternal reserves and post-reproduction survival, and all these traits are impacted by environmental conditions (Naulleau and Bonnet, 1996; Shine and Madsen, 1997; Bonnet *et al*., 1999, 2001a; Warner *et al*., 2008). Survival rate, fecundity and offspring quality, hence population viability, are thus determined by the sum of the physiological responses of individuals to environmental factors. Besides these idiosyncratic traits, most populations can be divided into major categories, notably sex and age. These categories exhibit physiological peculiarities that should also be considered to derive useful metrics. Overall, implementing selected ecophysiological measurements into population surveys is an asset to improve conservation actions (Fairbairn *et al*., 2007; Cooke and Suski, 2008; Dantzer *et al*., 2014).

Stress regulation is one of the major ecophysiological systems that allow individuals to adjust their behaviour, energy expenditure and reproductive effort to environmental constraints (Teixeira *et al*., 2007; Dickens *et al*., 2010). Glucocorticoid (GC) hormones are important effectors of the stress response; environmental stressors trigger an increase of GC concentrations that stimulates metabolism, vigilance and the mobilization of energetic resources. Thus, changes of GC blood concentrations have been widely used as key metrics of the adaptive capacities or health status in vertebrates (Wingfield and Romero, 2000; Romero and Wikelski, 2001; Kahn, 2006; Breuner *et al*., 2008). However, high GC concentrations can perturb sex steroid regulation and may negatively affect other functions (e.g. immunity; Charmandari *et al*., 2005; Breuner *et al*., 2008; Dantzer *et al*., 2014; Michel and Bonnet, 2014). Extreme and chronic stress responses can be detrimental to reproduction, survival and population viability, thereby revealing trade-offs between physiological functions (Sheriff *et al*., 2009; Zanette *et al*., 2011).

Nonetheless, many studies have failed to find clear relationships between GC concentrations, sex steroid concentrations and demography (Creel *et al*., 2009; Crespi *et al*., 2013; Dickens and Romero, 2013; Dantzer *et al*., 2014). Simple general patterns where elevated GC concentrations systematically correlate with population threats may not exist (Bonier *et al*., 2009; Boonstra, 2013). For example, although high chronic stress GC concentrations are interpreted as warning signs, a significant chronic/acute stress response may instead correlate positively with physiological stamina and, ultimately, with elevated reproductive performances (Dantzer *et al*., 2014). A flat stress response may indicate that the organism is exhausted and incapable of reacting to environmental factors rather than being unstressed (Dickens *et al*., 2010). In contrast, individuals exhibiting an extreme stress response would face difficulties in responding appropriately to environmental fluctuations. Either a lack of response (e.g. very low GC concentrations) or saturated responses (e.g. plateauing maximal CG concentrations or very high chronic concentrations relative to baseline values) suggest that physiological limits have been reached, with possible negative consequences. Therefore, determining the range of natural variation of physiological metrics (e.g. GC plasma concentrations) is a prerequisite to establish baselines in order to seize pertinent deviations that can be useful to field managers.

We measured the basal GC plasma concentrations of free-ranging Hermann's tortoises (*Testudo hermanni hermanni*) monitored by radio-tracking during 3 years. This endangered subspecies (IUCN, 2016) has faced a drastic reduction of its distribution range during recent decades, especially in continental areas (Livoreil, 2009; Bertolero *et al*., 2011). Many remaining populations occur at low densities, are fragile and are threatened by illegal harvesting, urbanization, closing of habitats and forest fires (Celse *et al*., 2014). Reintroduction (Bertolero *et al*., 2007; Livoreil, 2007; Bertolero, 2014) and, more recently, reinforcement programmes have been launched to bolster populations decimated by fire (Lepeigneul *et al*., 2014). Assessing the success of these translocations is important, but for comparative objectives, assessing the health status of resident individuals living in the remaining populations is needed. Although tortoises are robust animals that can afford harsh environmental conditions during prolonged periods, their adaptive capacities are not unlimited (Henen *et al*., 1998; Christopher *et al*., 1999). Consequently, an evaluation of the health status of tortoises sampled in contrasting and thus possibly challenging habitats (e.g. burned, closed) is important; therefore, we studied two populations in open and closed habitats, respectively.

Monitoring GC concentrations of individuals in different habitats represents a means to identify environmental stressors (Drake *et al*., 2012). Nonetheless, gathering CG concentrations in isolation is poorly informative because contrasted GC concentration profiles do not necessarily translate into contrasting demographic response. Taking into account a panel of traits is required to provide a better evaluation of threats to populations (Christopher *et al*., 1999; Cooke *et al*., 2013; Dantzer *et al*., 2014). In tortoises, behaviours, body condition and several other physiological metrics (e.g. haematocrit) are notably useful to evaluate the health and reproductive status of individuals (Henen, 1997; Christopher *et al*., 1999; Marín *et al*., 2002; Nagy *et al*., 2002; Lagarde *et al*., 2003a, 2008; Loehr *et al*., 2007; Lecq *et al*., 2014). It is also important to consider seasons and genders. In chelonians, each sex exhibits specific timing of reproductive effort (e.g. vitellogenesis, egg laying, spermatogenesis) reflected by sex seasonal patterns of blood chemistry in free-ranging (Henen *et al*., 1998; Christopher *et al*., 1999; Ott *et al*., 2000; Longepierre, 2001; Huot-Daubremont *et al*., 2003; Lagarde *et al*., 2003a; Currylow *et al*., 2013; Bonnet *et al*., 2016a) and captive tortoises (Kölle *et al*., 2001; Scope *et al*., 2013; Andreani *et al*., 2014).

The objective of this study was to establish dynamic ecophysiological references in Hermann's tortoises, taking into account possible sex and time effects. Therefore, in addition to CG concentrations, we monitored movements, body condition and several haematological traits involved in various functions (e.g. plasma concentrations of glucose as an index for energy budget; triglycerides for vitellogenesis; osmolality for water balance; uric acid for excretion). Given that habitats, seasons and years are likely to influence physiology, we sampled both sexes during the main activity periods, during 2 or 3 years consecutively, in both open mosaic and dense closed habitats.

## Materials and methods

### Study species and field sites

This study is part of a Life program (2010–2014; LIFE08NAT/F/000475) that aims to set up practical actions for the conservation of the Hermann's tortoise (Celse *et al*., 2014). Previously abundant in south-eastern France, this sub-species has markedly declined during recent decades; relict continental populations persist in the Massif des Maures and adjacent plains (Livoreil, 2009; Bertolero *et al*., 2011). This tortoise exhibits typical life-history traits of terrestrial chelonians, including delayed maturity, low fecundity and low population turnover (Bertolero *et al*., 2011). Females are on average 12% larger than males (Bertolero *et al*., 2011). Emergence from hibernation usually occurs from mid-March to the beginning of April, and the active season ends in November. Hermann's tortoises are found in various habitats, notably mosaic landscapes that comprise small cultivated fields, meadows, bushy zones and closed forest areas. They exhibit a generalist diet (mostly herbivorous) and are philopatric (Calzolai and Chelazzi, 1991).

**Figure 1:**
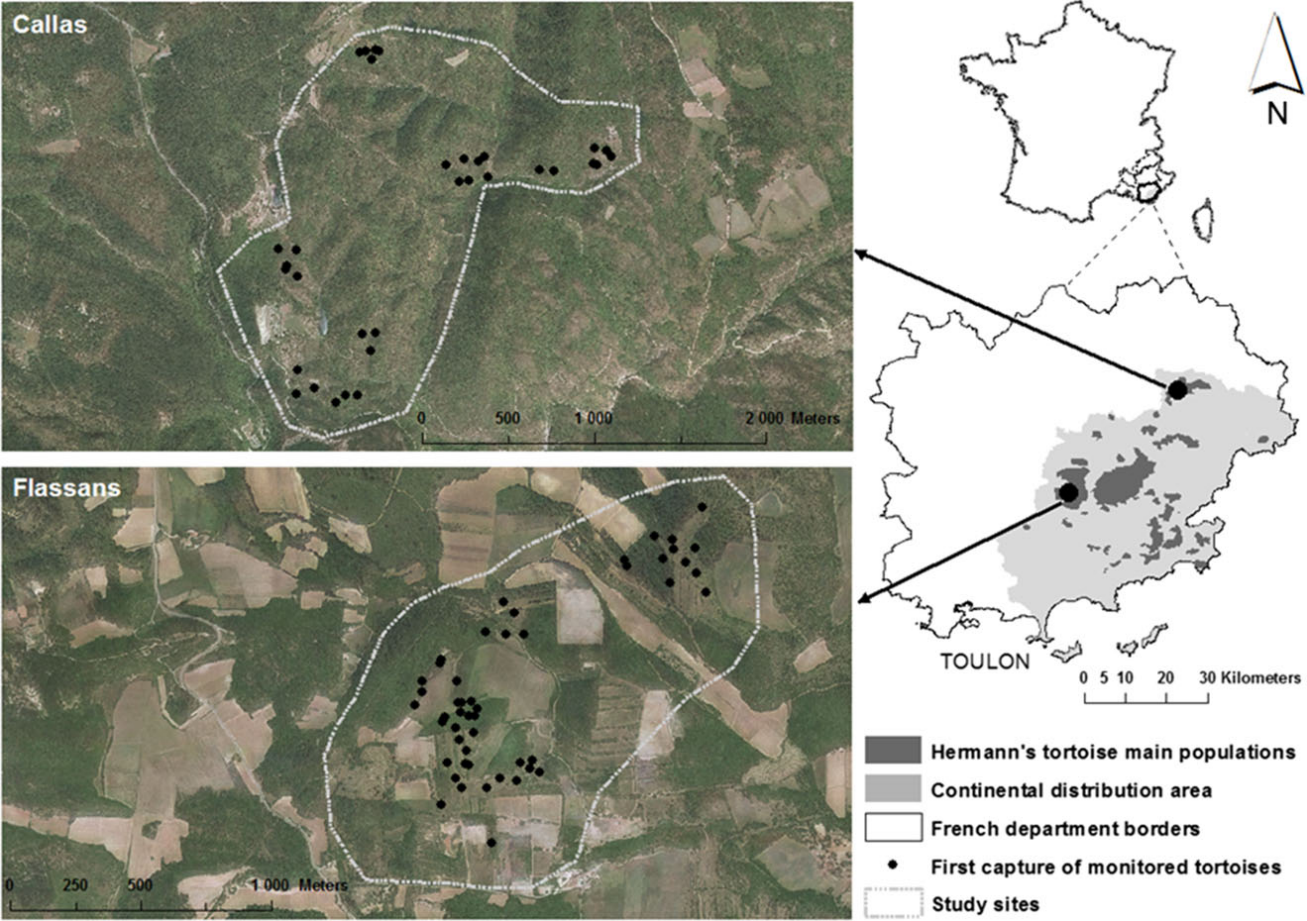
Location of the two study sites. The black dots indicate the initial position of the radio-tracked tortoises (all years pooled).

We studied two populations, Flassans and Callas, separated by ~38 km and seven roads (including a highway) and thus without any possibility for exchanges between them (Fig. 1). The respective habitats of these two populations are very different; they reflect variations across the current distribution range caused by human activities.

#### Flassans population

The Flassans population site (~50 hectares) is situated in the west region of the distribution range. Adult population density is estimated to be 4.2 tortoises/hectare J-M. B. and S. C. (unpublished data). This value is categorized as moderately high for French continental areas (Celse *et al*., 2014) but is low compared with less impacted areas (Bonnet *et al*., 2016b). This study site is relatively flat, with a mean elevation of 270 m (maximum 295 m). The geological substrate is calcareous. The habitat is open, characterized by small grassy meadows (29%), diverse and patchy shrub formations (29%, *Paliurus spina-christi*, *Cistus monspeliensis* and *Cistus albidus*), small woods (34%, *Quercus pubescens* and *Quercus ilex*), several small vineyards (7%), a temporary lake (1%) and numerous hedgerows. This study site is typical of traditionally managed agricultural areas and is considered to be favourable for reptiles, including tortoises.

#### Callas population

The Callas population (~225 hectares) is situated near the north-eastern limit of the distribution range. The adult population density is low, estimated to be 1.4 tortoises/hectare J-M. B. and S. C. (unpublished data). The study site is characterized by a hilly landscape, where elevation varies between 178 and 300 m. The geological substrate is siliceous. The sclerophyllous vegetation is mainly represented by a dense and close mixed forest with large trees (76%, *Quercus suber*, *Quercus pubescens* and *Pinus halepensis*), alternating with dense thick scrub vegetation (16%, mainly *Erica arborea*) and few partly open areas (8%, open patches populated by *Cistus albidus* and *Cistus monspeliensis*). This study site is typical of unmanaged areas where the habitat is progressively closing and is considered to be unfavourable for reptiles owing to a lack of basking and laying sites, shrub shelters and herbaceous layer (Todd and Andrews, 2008; Sirami *et al*., 2010).

Considering a broad geographical scale (100 km^2^) during the study period (2010–2012), mean temperatures in spring and summer were 17.7 and 21.5°C, respectively; mean precipitations were 86.2 and 28.6 mm, respectively (GES DISC, NASA, MERRA Monthly analyses). Climatic conditions varied among years (Table 1); probably without major differences between the two study sites considering the relatively short distance (<40 km) in terms of climatology [there is no major barrier (e.g. mountain) between sites].

**Table 1:** Mean temperature (in degrees Celsius) and total precipitation (in millimetres) during the three study periods according to the season

	Mean temperature (°C)		Precipitation (mm)	
	Spring	Summer	Spring	Summer
2010	17.55	23.30	452.2	72.4
2011	18.84	23.04	31.8	85.5
2012	18.23	24.18	221.3	55.8

### Population monitoring and radio-tracking

Free-ranging individuals were visually searched and captured after hibernation in April and early May. They were sexed (Djordjević *et al*., 2013), weighed [body mass (BM) ±1 g using a portable scale CL-Series; OHAUS], measured [shell length (SL) ±1 mm using callipers] and marked with a metallic clip (Livoreil, 2009). We selected tortoises with a shell length >120 mm; they were supposedly adult (Bertolero *et al*., 2011). Tortoises were fitted with an AVM-K16 transmitter glued onto the shell in early May. The tortoises were then immediately released. The transmitter plus resin represented <10% of individual body mass, a value assumed to be well tolerated in free-ranging tortoises (Lagarde *et al*., 2008). Each individual was located once a day, alternatively in the morning, around midday and in the afternoon. Coordinates were recorded using a Garmin GPS. Tortoises were weighed every 2 weeks, measured for SL, and blood sampled on two occasions during each active season, in spring and in summer. We selected these two periods because vitellogenesis occurs in spring, whereas intensive reproductive sexual behaviours are displayed by males in summer (Lagarde *et al*., 2003a).

In total, 82 different adult tortoises were monitored between 2010 and 2012. Eleven were monitored during 2 years and one was monitored during 3 years (total = 94 annual tortoise monitoring). Fifty tortoises were captured and radio-tracked at Flassans (23 in 2010, 22 in 2011 and 14 in 2012; eight monitored during more than 1 year). Thirty-two tortoises were studied at Callas (14 in 2010 and 21 in 2011; three monitored for more than 1 year). The overall sex ratio was balanced (42 females and 40 males). Daily displacements of radio-tracked tortoises were calculated as the mean distance travelled per day. There were measured during the spring season (before 21 June) to include vitellogenesis and the laying period and summer season (from the 21 June to 20 September), corresponding to the post-laying and main mating period.

### Body condition index

Body condition index (BCI) is an integrative metric that can involve various elements: body reserves, gut content, urinary bladder content and clutch mass (Bonnet *et al*., 2001b). It indicates the gross trophic and hydric status of individuals and is thus a key integrative parameter that responds to annual environmental fluctuations in tortoises (Lecq *et al*., 2014). It was estimated using the standardized residuals of the linear regression between the logarithm of BM and the logarithm of SL (Bonnet *et al*., 2001b).

### Blood sampling

To limit the possible stress effect during device fitting on blood parameters, we waited 15 days from initial capture before taking the first blood sample. Radio-tracked tortoises were sampled in spring (from 7 May to 5 June) and in summer (from 6 August to 21 September). We increased our sample size by incorporating tortoises opportunistically captured (OC) during radio-tracking sessions (*n* = 18 in Flassans and *n* = 6 in Callas). Some individuals were OC in a given year and radio-tracked another year. A total of 208 blood samples were collected from 96 individuals: 50 females and 46 males (two radio-tracked males from Callas were not blood sampled). Punctures were performed in the field before 12.00 h to limit the influence of daily variation. Samples were collected within 5 min to limit the impact of handling stress (Jessop *et al*., 2003; Drake *et al*., 2012; Bonnet *et al*., 2016a). Between 0.5 and 1.0 ml of blood was retrieved from the dorsal subcarapacial cervical plexus with a 25 or 26 gauge needle connected to a 1 ml syringe and transferred in a lithium heparinized tube. Blood samples were immediately placed on an ice bed in an icebox; they were transferred to the laboratory within 3–4 h after collection. We first measured the haematocrit (HCT; the percentage packed blood cell volume per unit volume of blood) by centrifuging blood in two capillaries (37 800 g, 3 min; Sigma 112 microcentrifuge). Haemodiluted samples detected by visual inspection during puncture (i.e. streaks of red liquid mixed up into a clear liquid; Bonnet *et al*., 2016a) contained substantial amounts of lymph (HCT <12%); they were discarded from statistical analysis. Samples were then centrifuged (10 000 rpm for 5 min), and the plasma was collected in small tubes and stored at −25°C until analysis.

### Hormonal assays

All assays could not be performed on several samples owing to limited amounts of blood retrieved, generating slight variations in sample sizes. The main GC in reptiles is corticosterone (CORT; concentrations usually expressed as nanograms per millilitre); plasma concentrations were assayed in the Centre d'Etudes Biologiques de Chizé (CEBC France) using radioimmunoassay (Bonnet *et al*., 2013, 2016a). The steroids were extracted from 40 μl of plasma using diethyl ether (mean extraction rate was of 97.3 ± 5.2%); the sensitivity of the assay was 1.9 pg/tube. Cross-reactions with other steroids were low (<0.1% for 11-deoxycorticosterone, cortisol, testosterone and androstenedione; 7% for compound S and progesterone). Intra- and interassay coefficients of variation remained <4%.

### Plasma metabolites and ion assays

The glycaemia (in milligrams per decilitre) was assayed directly in the field using a portable device, an Accu-Chek^®^ Performa blood glucose meter. Plasma concentrations of three circulating metabolites, triglycerides (in grams per litre), cholesterol (in grams per litre) and uric acid (in milligrams per litre), and of two ions, Na^+^ (in milliequivalents per litre) and K^+^ (in milliequivalents per litre), were measured at the BIO CONVERGENCE laboratory for medical analyses (Le Luc, France) using MODULAR de Roche automaton (ADVIA 2400 Siemens, Colorimetry, potentiometry). Osmolarity (in millimoles per litre) was calculated using the following formula: 2(Na^+^ + K^+^) (Dallwig et al. 2010; Guzman et al. 2011).

### Statistical analysis

Morphological data were logarithmically transformed to meet normality and homogeneity of variance assumptions. However, after transformation some variables were not normally distributed (corticosterone, uric acid and cholesterol concentrations); other variables (glycaemia, osmolality, haematocrit, triglyceride concentration, BCI and daily movements) were normally distributed. In addition, blood samples were repeated on some but not all individuals, resulting in a complex data set with several pseudo-replicates. Consequently, generalized linear mixed models with Gaussian or γ error and penalized quasi-likelihood estimation were used to assess the influence of sex and environmental variables on physiological markers (Bolker *et al*., 2009). Individual identity was added as a random factor. Each model included sex, year, season, site, BCI and SL as fixed variables. Initial models contained each variable and the first-order interaction between session and sex, session and year, and the interaction between sex and BCI; the interaction between sex and site was also integrated for the daily displacement analysis. Model selection was performed by backward elimination. Results with *P* < 0.05 were considered statistically significant, and tests were bidirectional. All analyses were performed with R version 2.13.1 (R version 2.13.1, 2011-07-08, © 2011, the R Foundation for Statistical Computing).

Many variables were measured, generating a possible risk of inflation in the presentation of the results. For conciseness, we retained a synthetic selection of the statistics, providing the final output from backward elimination summarized in Table 1. In addition, we provide more detailed results when appropriate in order to provide better focus on important issues (e.g. sex, season). Mean values ± SEM are indicated.

### Permits and ethical note

All procedures were performed in accordance with international regulations. Permits for population monitoring were issued by prefectural authorities on 13 January 2011. Ethical procedures were approved by the ethical committee COMETHEA (permit no. CE2013-6). No tortoise was injured during handling and blood sampling.

## Results

### Daily displacements

**Figure 2:**
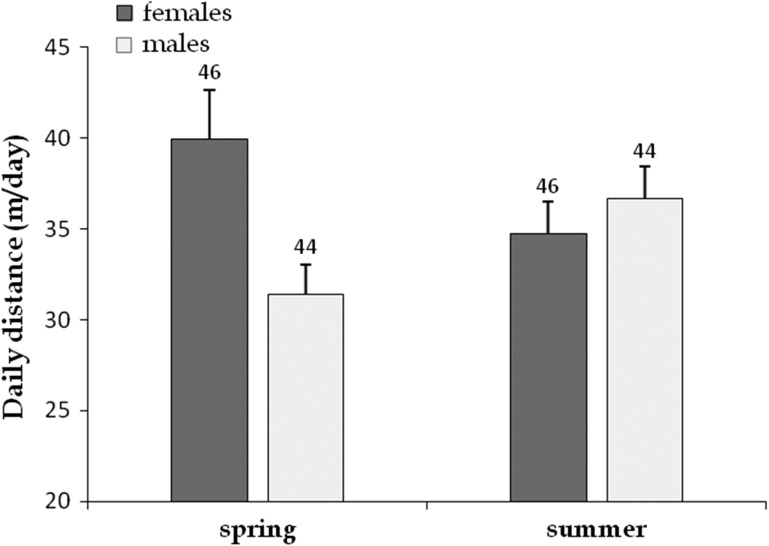
Effect of sex and season on the mean (+SEM) daily displacements of radio-tracked Hermann's tortoises. Two periods were considered: spring (left side), which corresponds to vitellogenesis and laying periods, and summer (right side), which corresponds to the main season of sexual activity in males. We found significant effects of sex and season, and a significant interaction between these factors (see Table 1).

**Figure 3:**
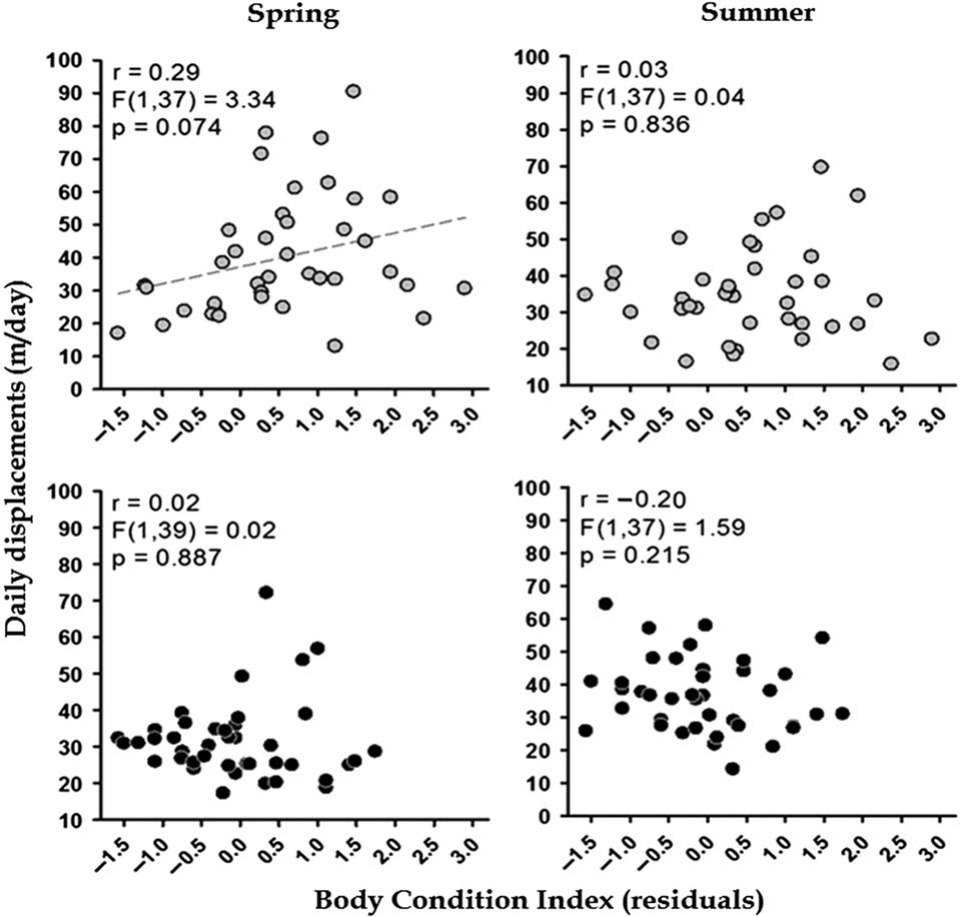
Relationship between body condition index and mean (±SEM) daily displacements of radio-tracked females (grey symbols; top panels) and males (black symbols; bottom panels). Individuals were monitored in spring (left panels) and in summer (right panels). Body condition index was calculated at the beginning of each season. The dashed line indicates a correlation close to statistical significance.

We found significant effects of sex, study site, season, body size and body condition on mean daily displacements, with several significant interactions among these factors but without an effect of year (Table 2). Greater spring movements in females compared with males (39.9 ± 2.7 vs. 31.4 ± 1.7 m/day, respectively; restricting the analysis to spring, *F*_1, 72_ = 7.11, *P* < 0.001) are likely to have induced the interaction between sex and season (Fig. 2). The situation was partly reversed in summer, with no difference between the sexes (34.7 ± 1.8 in females vs. 36.7 ± 1.8 m/day in males; *F*_1, 69_ = 3.32, *P* = 0.07; Fig. 2). In spring, BCI weakly influenced daily displacements in females (Fig. 3) but not in males; no effect of BCI was observed in summer. Mean daily movements were slightly albeit significantly higher in Callas than in Flassans (40.4 ± 1.9 and 33.2 ± 1.2 m/day, respectively; Table 2).

**Table 2:** Effect of temporal (season, year), spatial (site) and individual parameters (sex, body condition index, shell length) on displacements, body condition and a set of seven physiological markers

Dependent variable	Effect	d.f.	*t*-value	*P*-value
Daily displacements (*n* = 157)	Intercept	85	−0.03	0.975
	Sex	65	2.28	**0.026**
	Site	65	−2.54	**0.014**
	Season	85	2.00	**0.049**
	SL	65	2.24	**0.028**
	BCI	65	2.35	**0.022**
	Sex*BCI	65	−2.01	**0.048**
	Sex*Season	85	−2.73	**0.008**
Body condition index (*n* = 209)	Intercept	114	3.22	**0.002**
	Sex	91	−2.94	**0.004**
	Season	114	−0.96	0.338
	Sex*Season	114	−2.94	**0.004**
Haematocrit (*n* = 157)	Intercept	82	1.16	0.248
	Sex	82	5.08	**<0.001**
	Year 2010–11	67	−4.41	**<0.001**
	Year 2010–12	67	−0.56	0.579
	Year 2011–12	67	3.46	**0.001**
	Season	67	−2.88	**0.005**
	SL	67	2.93	**0.005**
	Year 2010–11*Season	67	5.79	**<0.001**
	Year 2010–12*Season	67	1.79	0.077
	Year 2011–12*Season	67	−2.95	**0.004**
Corticosterone (*n* = 196)	Intercept	96	7.68	**<0.001**
	Sex	92	−3.23	**0.002**
	Year 2010–11	96	4.48	**<0.001**
	Year 2010–12	96	0.13	0.898
	Year 2011–12	96	−3.89	**<0.001**
	Season	96	6.70	**<0.001**
	Sex*Season	96	−6.65	**<0.001**
	Year 2010–11*Season	96	3.77	**<0.001**
	Year 2010–12*Season	96	−1.42	0.157
	Year 2011–12*Season	96	2.39	**0.019**
Glycaemia (*n* = 128)	Intercept	65	19.78	**<0.001**
	Sex	65	−3.12	**0.003**
	Year 2011–12	61	3.66	**<0.001**
	Season	61	−1.63	0.107
	Sex*Season	61	4.22	**<0.001**
Triglyceride (*n* = 122)	Intercept	78	16.11	**<0.001**
	Sex	78	−12.65	**<0.001**
	Year 2010–11	40	2.90	**0.006**
	Year 2010–12	40	2.94	**0.005**
	Year 2011–12	40	0.53	0.601
Cholesterol (*n* = 120)	Intercept	77	9.25	**<0.001**
	Sex	77	6.60	**<0.001**
	Year 2010–11	39	−3.04	**0.004**
	Year 2010–12	39	−3.30	**0.002**
	Year 2011–12	39	−0.75	0.459
Uric acid (*n* = 177)	Intercept	92	9.00	**<0.001**
	Year 2010–11	79	3.31	**0.001**
	Year 2010–12	79	1.92	0.058
	Year 2011–12	79	−0.50	0.619
	Season	79	4.56	**<0.001**
	Year 2010–11*Season	79	−2.12	**0.037**
	Year 2010–12*Season	79	−1.99	0.050
	Year 2011–12*Season	79	−0.38	0.703
Osmolarity (*n* = 144)	Intercept	86	67.98	**<0.001**
	Sex	86	3.03	**0.003**
	Year 2010–11	52	0.74	0.463
	Year 2010–12	52	5.11	**<0.001**
	Year 2011–12	52	5.83	**<0.001**
	Season	52	4.46	**<0.001**
	Sex*Season	52	−2.64	**0.011**

BCI, body condition index; SL, shell length. Significant *P*-values are indicated in bold.

### Body condition index

**Figure 4:**
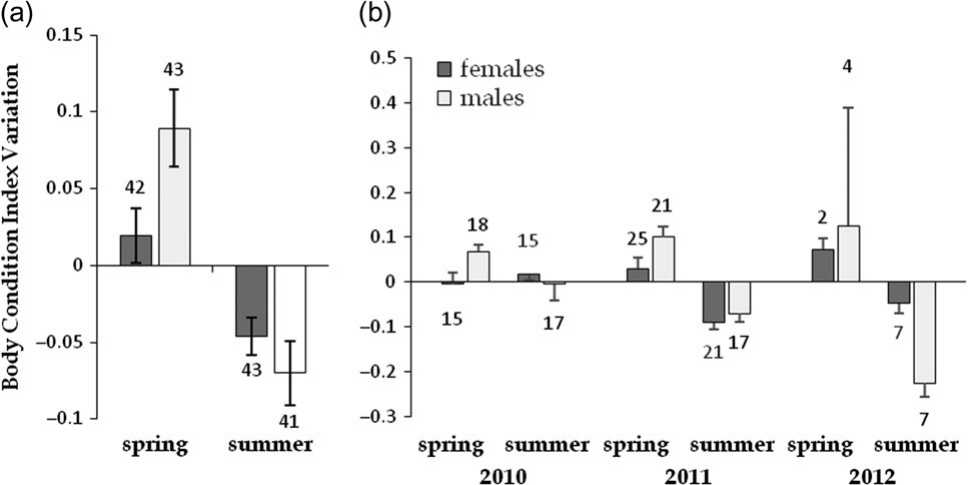
Effect of sex and season (**a**) and sex, season and year (**b**) on the mean variations of body condition index (±SEM) in radio-tracked Hermann's tortoises. The numbers above or below the bars indicate sample size. We found significant effects of sex, season and their interactions (see Table 2).

Body size ranged from 120 to 203 mm in females and from 116 to 162 mm in males. Body mass ranged from 433 to 1597 g in females and from 316 to 877 g in males. Females were larger than males; mean body mass and mean shell length were 855 ± 34 g and 521 ± 14 cm vs. 160 ± 35 g and 137 ± 15 cm, respectively (ANOVA with sex as the factor and log-BM or log-SL as the dependent variables, respectively: *F*_1, 80_ = 88.46, *P* < 0.01 and *F*_1, 79_ = 58.51, *P* < 0.01). The BCI varied significantly between sexes, with a significant interaction with season (Table 2). Mean BCI increased in spring (+0.05 ± 0.02, sexes and years pooled) and decreased in summer (−0.06 ± 0.01). Although the generalized linear mixed model analysis did not reveal a significant effect of year, we nonetheless examined this factor further (Fig. 4b). A consistent marked pattern was observed during 3 years in males, with an increase of BCI in spring and a decrease in summer; this pattern was significantly less pronounced and visible in females (Fig. 4a). Therefore, the trend with spring increase vs. summer decrease of BCI was not attributable to a peculiar year or driven by one sex only.

### Haematological traits

In order to provide references and to detect outliers (revealing possible disorders), we first present descriptive statistics using the full data set (i.e. including pseudo-replicates; Table 3). All parameters examined showed important ranges of variation, and few outliers were detected (Table 3). Next, we examine individual, temporal and site effects on each haematological metric examined (Table 2). As a common output, neither the site nor the BCI had a significant effect on the haematological traits (Table 2). Below, we review the main effect detected on each blood trait.

**Table 3:** Reference levels for haematological metrics of *Testudo hermanni hermanni*

Parameter	Mean	*n*	SD	Minimum	Maximum	95% Confidence interal	Outlier
Haematocrit (%)	22.05	161	6.31	12	38	21.08–23.03	No
Female	19.98	79	5.51	12	32	18.77–21.20	
Male	24.05	82	6.41	12	38	22.66–25.43	
Corticosterone (ng/ml)	1.85	196	1.60	0.15	8.64	1.63–2.08	*n* = 5, >5.45
Female	0.96	100	0.90	0.15	5.25	0.78–1.13	
Male	2.79	96	1.64	0.38	8.64	2.47–3.13	
Glycaemia (mg/dl)	68.03	131	20.8	14	129	64.46–71.59	*n* = 1, >110
Female	68.04	68	22.78	14	129	62.63–73.46	
Male	68.01	63	18.62	16	108	63.42–72.61	
Triglyceride (g/l)	2.24	122	2.05	0.00	7.52	1.88–2.60	No
Female	3.84	61	1.74	0.50	7.52	3.40–4.28	
Male	0.64	61	0.50	0	2.55	0.52–0.77	
Cholesterol (g/l)	1.28	120	0.70	0.11	3.83	1.16–1.41	*n* = 3, >2.87
Female	1.65	61	0.65	0.49	3.34	1.49–1.82	
Male	0.89	59	0.51	0.11	3.83	0.76–1.02	
Uric acid (mg/l)	15.90	177	12.25	0.2	68	14.10–17.71	*n* = 4, >42
Female	15.89	93	13.09	1	68	13.23–18.55	
Male	15.91	84	11.34	0.2	60	13.49–17.71	
Omsolarity (mmol/l)	272.25	144	15.67	241.2	328.4	269.69–274.81	*n* = 8, >289.4
Female	270.12	77	14.35	251.2	328.4	266.91–273.32	
Male	274.70	67	16.84	241.2	326	269.69–274.81	

Removing outliers from the data set markedly affected reference value that might be used as ecophysiological references for the species.

Mean values of HCT exhibited a complex pattern. They were significantly influenced by sex, size, year and season, with an interaction between year and season (Table 2). The females exhibited lower HCT compared with males (19.98 ± 0.6 vs. 24.05 ± 0.7). Other effects were either very weak (correlation between HCT and body size: *r*^2^ = 0.02) or inconsistent regarding explanatory variables.

**Figure 5:**
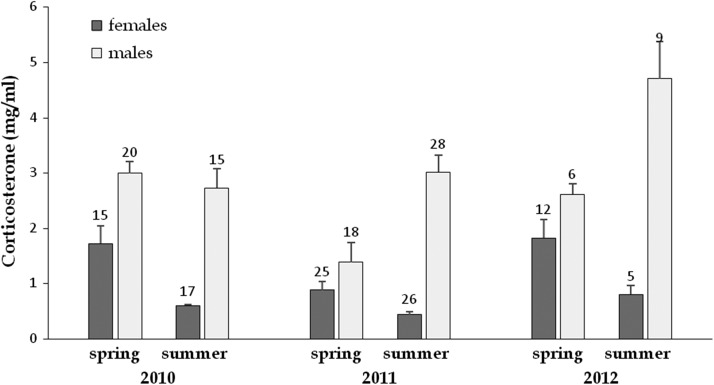
Annual and seasonal variations of plasma corticosterone concentration (shown as mean values + SEM) in Hermann's tortoises. We found significant effects of year and season, and significant interactions between sex and season and between year and season (see Table 2). The numbers above the bars indicate sample size.

Mean values of CORT were influenced by most of the factors tested, with significant interactions among them (Table 2), resulting in complex effects. However, a close inspection of the data revealed a consistent broad pattern. Females systematically exhibited lower CORT compared with males, with almost no overlapping of the values between the sexes in summer (Fig. 5). In females, CORT decreased systematically from spring to summer (years pooled: spring CORT = 1.34 ± 0.2 ng/ml,; summer CORT = 0.54 ± 0.04 ng/ml), whereas on average the opposite pattern was observed in males (spring CORT = 2.28 ± 0.2 ng/ml; summer CORT = 3.22 ± 0.2 ng/ml). Pooling sexes and seasons, CORT was significantly higher in 2010 (2.05 ± 0.2 ng/ml) and 2012 (2.65 ± 0.3 ng/ml) than in 2011 (1.47 ± 0.2 mg/ml).

**Figure 6:**
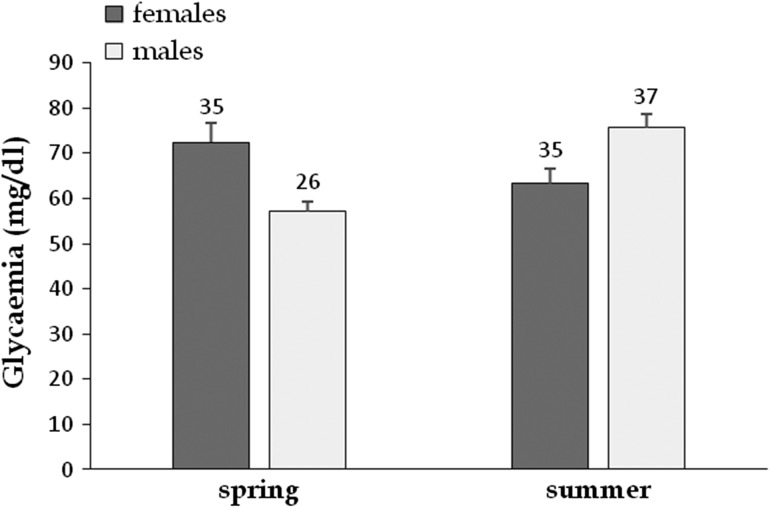
Effect of sex and season on glycaemia (shown as mean values + SEM) of radio-tracked Hermann's tortoises. Two periods were considered; spring corresponds to vitellogenesis and laying periods, whereas summer corresponds to intensive male sexual activity. The numbers above the bars indicate sample size.

Mean glycaemia was significantly influenced by sex, year and an interaction between sex and season (Table 2). In females, glycaemia decreased from spring to summer (72.51 ± 4.2 and 63.30 ± 3.4 mg/dl, respectively; Fig. 6); the opposite pattern was observed in males (57.07 ± 2.4 and 75.70 ± 3.1 mg/dl, respectively). Glycaemia was higher in 2012 (78.45 ± 3.5 mg/dl) than in 2011 (64.52 ± 2.0 mg/dl).

Mean plasma concentrations of triglycerides varied significantly between sexes and among years (Table 2). Triglycerides were significantly higher in females (3.84 ± 0.2 g/l) than in males (0.64 ± 0.1 g/l). They were significantly lower in 2010 than in 2011 and 2012 (1.35 ± 0.2, 2.32 ± 0.2 and 2.52 ± 0.4 g/l, respectively).

Mean plasma concentrations of cholesterol varied significantly between sexes and among years (Table 2). Cholesterol concentrations were significantly higher in females (1.65 ± 0.08 g/l) than in males (0.89 ± 0.07 g/l). They were higher in 2010 than in 2011 and 2012 (1.53 ± 0.06, 1.24 ± 0.1 and 1.43 ± 0.10 g/l, respectively).

Mean concentrations of uric acid were mainly impacted by season and year (Table 2). Mean values were almost 2-fold higher in spring than in summer (19.3 ± 1.4 vs. 12.0 ± 0.9 mg/l). Uric acid concentrations differed among years (in 2010, 2011 and 2012: 18.5 ± 1.9, 13.7 ± 1.0 and 15.8 ± 1.4 mg/l, respectively).

**Figure 7:**
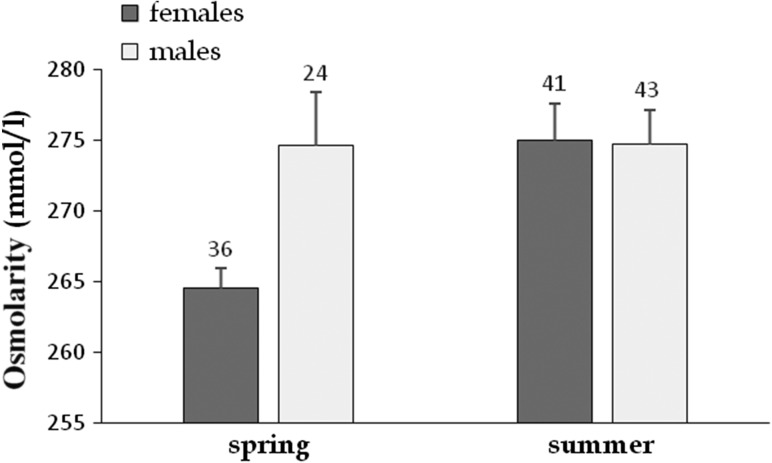
Effect of sex and season on Osmolarity (shown as mean values + SEM) of radio-tracked Hermann's tortoises. Two periods were considered; spring corresponds to vitellogenesis and laying periods, whereas summer corresponds to intensive male sexual activity. The numbers above the bars indicate sample size.

Mean Osmolarity was mainly influenced by season, year and sex (Table 2). Osmolarity was significantly higher in males than in females (274.70 ± 2.1 vs. 270.12 ± 1.6 mmol/l, respectively). It was significantly higher in 2012 than in 2010 and 2011 (283.67 ± 4.3, 270.95 ± 1.3 and 268.17 ± 1.2 mmol/l, respectively); it varied between seasons in females (spring 264.55 ± 1.4 mmol/l; summer 275.01.0 ± 2.6 mmol/l; Fig. 7) but not in males (spring 274.65 ± 3.8 mmol/l; summer 274.73 ± 2.4 mmol/l).

## Discussion

Setting up simple field technique(s) to determine sex-specific baselines along with the range of variations of several major ecophysiological metrics may assist field managers to monitor the health status of tortoises. Chelonians are robust organisms that can survive harsh conditions, but they may not reproduce at a sufficient rate for population viability if, for example, vitellogenesis and thus egg production is perturbed (Turner *et al*., 1986). Detection of underlying physiological disorders requires reference values gathered in normally functioning individuals in various conditions.

Obtaining these values from free-ranging tortoises is relatively simple. Tortoises are slow-moving animals tolerant to handling, to the electronic device glued onto their back and to repeated blood sampling (Lagarde *et al*., 2008; X.B., unpublished observations). Thus, individuals can be monitored on a regular basis and their global status (BCI, behaviours and blood parameters) can be measured accurately. Possible physiological disorders (e.g. decreasing BCI, chronic high CORT) caused by translocation and/or degraded habitats can be detected early, possibly prompting interventions (e.g. removal of individuals from unsuitable habitats). More subtle effects, such as a lack of elevation of plasma concentrations of cholesterol and triglycerides in spring in females (i.e. an index of vitellogenesis), would reveal reproductive disorders, motivating further investigations.

In practice, however, interpretion of individual and mean values can be tricky because of strong variations in most behavioural and physiological traits of chelonians. For example, a very low glycaemia (<0.4 g/l) can be lethal in endotherm vertebrates (Chajek-Shaul *et al*., 1990), whereas it may simply reflect non-pathological natural fluctuations in ectothermic reptiles, where variations are driven by ambient temperatures and reproductive effort (Bonnet and Naulleau, 1993). Consequently, although a very low glycaemia may well suggest serious health problems in an active female during vitellogenesis, it should be considered as normal in a resting and cold female sampled in late summer. Considering a set of metrics and environmental factors is thus important to assist the diagnosis of health status in organisms that display a very flexible physiology, such as reptiles (in less flexible organisms, such as birds, for instance, any deviation of natraemia or body temperature may represent a warning sign). For instance, a counterintuitive strong reduction of albuminuria concomitant with an increase in total plasma proteins can be explained by a shift of liver function during vitellogenesis in reptiles (Bonnet *et al*., 1994), but this does not indicate a pathological state, as would be the case if ‘endothermic references were used (Hill *et al*., 1977; Lumeij, 1987; Cerón *et al*., 2005; Roche *et al*., 2008). It is thus important to describe the range of physiological variations in free-ranging and healthy individuals in the course of their normal activity in order to derive useful reference values.

This study combined various metrics recorded in adult female and male tortoises monitored during several years. None of the individuals presented any sign of disorder, exhibited a marked decrease of BCI or displayed unusual behaviours. Many matings were observed (*n* = 82). Several females were observed while laying their eggs (*n* = 4 individuals; one female laid two clutches), and we found 31 nests in the field. Considering that witnessing laying females and finding nests represent rare events in the field for this species, we crudely estimate that reproductive rate was normal. On average, tortoises travelled 30–40 m/day, covering greater distances in the closed habitat (Callas) compared with open mosaic habitat (Flassans) that is considered to be more favourable for Hermann's tortoises (Couturier *et al*., 2014). Perhaps the closed habitat forced tortoises to move more often between shelters, foraging and basking spots. Whatever the case, these values fall within the range of variations for *Testudo* species living in relatively similar habitats [15–30 m/day for *T. h. hermanni* in Italy, Chelazzi and Francisci (1979); 32 m/day for *T. h. boetgerri* in Romania, Rozylowicz and Popescu (2013); 20–120 m/day for *T. graeca* in Spain, Díaz-Paniagua *et al*. (1995)]. The sex pattern with more mobile females during vitellogenesis and laying periods was also documented (Díaz-Paniagua *et al*., 1995). In markedly different habitats, other patterns may emerge; larger daily displacements for males during the mating period (160 ± 40 vs. 67 ± 28 m/day during the post-mating period), with lower mean daily displacements and a larger home range in females have been recorded in the steppe tortoise, *Testudo horsfieldi* (Lagarde *et al*., 2002, 2003b). Focusing on Mediterranean habitats, the daily displacements we recorded are representative of tortoises in the course of their usual daily activity.

As expected for this species, females were larger compared with males (Willemsen and Hailey, 2003; Djordjević *et al*., 2013). Body condition increased in spring and decreased in summer, probably because feeding activity culminates in spring (Calzolai and Chelazzi, 1991; Rugiero and Luiselli, 2006; Christopher *et al*., 1999; Nagy *et al*., 2002). Yet, dehydration may participate in the summer decrease of BCI. In addition, males tend to feed less during the mating season in summer. These results are typical for tortoises (Henen, 1997; Loehr *et al*., 2007) and they confirm that we monitored ‘normal’ individuals exposed to natural environmental fluctuations. Overall, we believe that our blood samples did not include sick or abnormal individuals and that the range of variations of the parameters measured reflect sex-specific responses to annual fluctuations of normally breeding, free-ranging Hermann's tortoises living in contrasting habitats. Thus, Table 3 provides reference values that can be used to gauge to what extent any individual may deviate from the expected range of variations. However, in order to interpret these values correctly, several factors should be considered. We generally found strong effects of sex, season and year, and interactions among them.

The most consistent effects involved sex and season. Corticosterone concentrations were notably higher in males than in females; a result in accordance with previous studies (Schramm *et al*., 1999; Lance *et al*., 2001; Drake *et al*., 2012; Selman *et al*., 2012). Corticosterone decreased during the active season in females but increased in males; a similar pattern was observed for glycaemia. Thus, the present study corroborates the notion that CORT concentration is involved in the mobilization of energy stores, such as glucose (Sapolsky *et al*., 2000; Moore and Jessop, 2003; Crespi *et al*., 2013). The sex–season interaction may mirror the sexual seasonal difference of reproductive effort; vitellogenesis in spring vs. mate searching in summer. Plasma concentrations of the main sex steroids also vary between sexes and seasons in chelonians (Schramm *et al*., 1999; Ott *et al*., 2000; Lance *et al*., 2001; Huot-Daubremont *et al*., 2003; Sereau *et al*., 2010; Currylow *et al*., 2013; Wack *et al*., 2008). If CORT (basal concentrations) supports the mobilization of resources for reproduction, then parallel seasonal fluctuations of plasma concentrations of estradiol and testosterone (in females and males, respectively) and CORT should occur (Crespi *et al*., 2013). As expected, females exhibited higher concentrations of plasma lipids (triglycerides and cholesterol) than males. Indeed, lipids are typical markers of vitellogenesis (Bonnet *et al*., 1994; Duggan *et al*., 2001; Lagarde *et al*., 2003a). Moreover, it has been shown that CORT enhances food intake and daily activity (Cote *et al*., 2006). We observed similar pattern of variations between sex and season in CORT, glycaemia and daily movement, with an overall decrease for females between spring and summer and an overall increase for males. Uric acid concentrations were higher in spring than summer. The opposite pattern was found for the osmolality, a trait mainly influenced by a low osmolality of the females in spring (Fig. 7), followed by an elevation in summer, perhaps owing to low precipitation. Osmolality and HCT were higher in males than in females, possibly because of their higher surface/body mass ratio that promotes dehydration. High HCT might be related to male velocity (Bonnet *et al*., 2001b). Substantial water lost during egg laying may also impact female hydration level (Osmolarity and HCT) because eggs contain important amounts of water at deposition (Tracy *et al*., 1978; Turner *et al*., 1986). Further studies are required to clarify these issues in tortoises.

Annual variations in interaction with seasons represent another important source of fluctuations of the blood parameters measured; notably, considering uric acid, CORT and HCT. Annual changes in food and water availability associated with fluctuating ambient temperatures influence almost all life-history traits in ectotherms, including metabolism and haematological parameters (Packard, 1991). Other factors (e.g. food availability, diet, drinking sites, shelter abundance, predators) and interactions among individuals may also influence physiological traits. Most of these factors were present in our two study sites and applied in a peculiar way to each individual (e.g. predators killed several tortoises in our two study sites and probably threatened others, but not all), and thus our metrics already incorporate these sources of variation. However, monitoring individuals during extreme climatic events (e.g. prolonged drought) would be helpful to calibrate ecophysiological references better. Our 3 years of study, with the alternation of springs and summers, nonetheless offered substantial variations. But we probably missed extreme and thus very informative events; opportunistic assessments would be helpful to examine whether strong droughts are detrimental or not (e.g. individuals may simply aestivate; Lagarde *et al*., 2002). Extreme events (e.g. repeated fires, prolonged droughts) are likely to be very important in terms of physiological response and population viability.

### Conclusions

Our results suggest that, in order to be exploitable by field researchers, ranges of fluctuations of ecophysiological metrics should be considered for each sex and season (Table 3). The set of parameters measured, encompassing behaviours, body condition and various haematological traits, suggest that spring is a crucial period for females, whereas summer is the most demanding season for males. This sex difference may guide the selection of distinct and supposedly appropriate periods to set up field actions, such as translocations. Importantly, the reference levels for haematological metrics provided in Table 3 should allow rapid and simple monitoring of the health status of tortoises in the future, both during field experiments and to survey remaining populations. The development of portable devices facilitates this type of investigation (Stoot *et al*., 2014), but interpretations rely on accurate baselines.
